# Pharmacogenetic Analysis of Variants in IL-6 Signaling and Response to Modern Therapeutic Approaches in Greek Patients with Atopic Dermatitis

**DOI:** 10.3390/genes17050575

**Published:** 2026-05-18

**Authors:** Dimitra Triantafillidi, Vasiliki Tziouvara, Alexandros Pontikas, Adam Akritidis, Charalabos Antonatos, Aikaterini Zacharopoulou, Aikaterini Tsiogka, Ileana-Afroditi Kleidona, Katerina Grafanaki, Alexandra Chrysospathi, Niki Ntavari, Elli Kampra, Sophia Georgiou, Efterpi Zafiriou, Stamatis Gregoriou, Yiannis Vasilopoulos

**Affiliations:** 1 Laboratory of Genetics, Section of Genetics, Cell Biology and Development, Department of Biology, University of Patras, 26504 Patras, Greece; dimitra.eirini.triantafyllidi@gmail.com (D.T.);; 2 Department of Dermatology-Venereology, Faculty of Medicine, Andreas Sygros Hospital, National and Kapodistrian University of Athens, 16121 Athens, Greece; 3 Department of Dermatology-Venereology, School of Medicine, University of Patras, 26504 Patras, Greece; 4 Department of Dermatology, University General Hospital Larissa, University of Thessaly, 41334 Larissa, Greece

**Keywords:** atopic dermatitis, eczema, pharmacogenetics, dupilumab, upadacitinib, response to therapy

## Abstract

Background/Objectives: We conducted the first pharmacogenetic investigation of atopic dermatitis in a cohort of 43 Greek patients, focusing on key variants within the IL6/JAK/STAT signaling axis, a pathway central to inflammation and therapeutic targeting. Methods: Patients receiving dupilumab, JAK inhibitors, or topical corticosteroids were prospectively evaluated, with treatment response assessed by changes in the Eczema Area and Severity Index over four months. Targeted genotyping of *IL6R* rs2228145 A>C, *JAK1* rs2780815 T>G, and *TRAF3* rs12147254 G>A were performed using PCR-RFLP. Results: Across the full cohort, no robust pharmacogenetic effects were detected, while baseline disease severity was the strongest predictor of absolute clinical improvement. However, stratified analyses revealed a significant association between the *IL6R* rs2228145 minor allele and reduced upadacitinib response (*p*-value = 0.026). Consistently, the same variant demonstrated a nominal association with reduced likelihood of achieving ≥75% improvement (*p* = 0.065). Conclusions: Although limited by sample size, these findings suggest potential treatment-specific pharmacogenetic effects within the IL6 pathway, supporting further investigation in larger cohorts to inform personalized therapeutic strategies in eczema.

## 1. Introduction

Atopic dermatitis (AD), also known as atopic eczema, is a chronic pruritic, inflammatory skin disease frequently associated with other atopic syndromes including food allergy, allergic rhinitis and asthma. It represents the most common inflammatory skin disease in childhood, affecting approximately 15–20% of children and, to a lesser extent, 1–3% of adults worldwide. Clinically, AD is characterized by recurrent episodes of pruritus, erythema, edema, xerosis, crusting, and lichenification. The clinical presentation varies with age of onset and disease stage, reflecting the heterogeneous nature of the disorder [1].

The pathophysiology of AD is highly complex, involving genetic susceptibility, immune dysregulation, epidermal barrier dysfunction, and environmental triggers. A strong genetic component has been demonstrated through family aggregation and twin studies. The presence of any atopic disease in a parent increases the risk of AD in offspring by approximately 1.5-fold, while parental AD increases this risk by 3–5-fold. Concordance rates of AD range from 72 to 86% in monozygotic twins compared with 21 to 23% in dizygotic twins, suggesting a heritability estimate of approximately 70 to 80% [2]. Among the most prominent genetic components are loss-of-function (null) mutations in the filaggrin gene (*FLG*), observed in approximately half of patients with moderate-to-severe AD [3]. This dysfunction results in increased transepidermal water loss and facilitates the penetration of environmental allergens and irritants into the skin, thereby promoting inflammatory responses. In addition to barrier dysfunction, genetic variation affecting immune signaling pathways plays a central role in disease pathogenesis. Several susceptibility loci have been identified in genes regulating cytokine signaling and immune responses, including regions on the 5q31-33 chromosomal region associated with proinflammatory cytokine production, maintenance of the allergic reaction during later chronic stages and, in general, disease progression [4]. Nevertheless, genetic predisposition alone does not fully explain disease onset, since environmental factors, such as allergens and air pollution, can interact with host genetic factors to influence disease onset and severity. Epidermal barrier dysfunction, largely driven by *FLG* loss-of-function mutations, further amplifies this interaction by enabling enhanced penetration of external agents into the skin [5].

This complex background is further reflected upon the diversity of biological pathways that contribute to disease pathogenesis. Among these, JAK/STAT signaling holds a central role in mediating inflammatory responses at both acute and chronic AD stages, regulating epidermal barrier dysfunction and modulating pruritus transduction [6]. This inflammatory cascade is initiated by the binding of interleukin (IL)6 to its receptor, IL6R, which is in a complex with the cell surface signaling receptor glycoprotein 130 (gp130). This binding results in the activation of Janus-activated kinase 1 (JAK1) and the subsequent phosphorylation of gp130 [7]. Phosphorylated gp130 then recruits the signal transducer and activator of transcription 3 (STAT3), which is phosphorylated and activated by JAK1. Activated STAT3 translocates into the nucleus to promote target gene expression. Naturally, this signaling axis is further controlled by intracellular negative regulators; protein tyrosine phosphatase non-receptor type 22 (PTPN22) can be recruited via TNF receptor-associated factor 3 (TRAF3) to the receptor complex, thereby attenuating IL6-dependent signaling through dephosphorylation of JAK1 and STAT3 [8]. Both *IL6R* and *TRAF3* genes have been previously identified as major risk genes for AD, prioritized as the highest-scoring genes in their respective loci [9]. Similar genetic evidence has also been reported for JAK1 [10], standing out as a master regulator in multiple common inflammatory diseases, mediating signaling for at least 28 different pro-inflammatory cytokines—nearly half of all ligands employing the JAK-STAT pathway [11].

Recognition of the central role of cytokine-mediated signaling in AD has reshaped therapeutic strategies through the development of targeted immunomodulatory therapies [12]. The monoclonal antibody dupilumab is the first approved targeted biological therapy for moderate-to-severe AD in infants, children and adults [13]. By binding to the interleukin 4 receptor a, dupilumab inhibits the IL4/IL13 signaling pathway—two central mediators of type 2 inflammation during AD acute phase. In addition to biologics, the introduction of small molecule therapies such as oral JAK inhibitors has provided a novel treatment modality, addressing limitations of existing therapies and enabling more individualized management of AD.

Despite the promising efficacy of modern therapies, there is considerable inter-individual variability in treatment response, with patients experiencing partial or even no response, as well as adverse effects that necessitate predictive biomarker identification that could guide treatment selection [14]. However, little effort has been made to identify reliable treatment response biomarkers for AD in general. A recent systematic review evaluated 37 different records investigating potential biomarkers associated with response to systemic therapies and adverse effects, reporting inconsistent findings across established AD-related markers such as serum IgE, eosinophil level and circulating cytokines [15,16]. Lactate dehydrogenase (LDH) was the only reliable marker that demonstrated some predictive potential, although evidence remains scarce from two studies [17,18]. Importantly, most studies showed poor consensus regarding clinical and molecular endpoints, lacking evaluation of any biomarker investigation at the genetic level. Indeed, pharmacogenetics, that is, the study of the impact of genetic variations on drug response, is a key pillar of personalized medicine [19]. Variants including single nucleotide polymorphisms (SNPs), copy number variations (CNVs), and insertions/deletions in genes involved in cytokine signaling, receptor function, or drug metabolism, can influence both the efficacy and safety of therapies [20]. Our group has consistently evaluated pharmacogenetic associations in psoriasis, another inflammatory skin disease with a T helper (Th)1/17-skewed inflammatory response, uncovering potential response biomarkers to biologic therapies including anti-TNF [21,22], as well as conventional approaches such as cyclosporine [23,24]. To date, no comparable pharmacogenetic studies have been conducted in AD.

In the present study, we performed the first pharmacogenetic investigation of AD in a cohort of 43 Greek patients. We focused on genetic variation within the IL6 signaling axis of the JAK/STAT pathway due to its established role in chronic inflammatory responses, transition from acute to chronic inflammation and its interaction with cytokine pathways targeted by modern therapies. We evaluated the role of the *TRAF3* rs12147254 G>A, *JAK1* rs2780815 T>G and *IL6R* rs2228145 A>C polymorphisms in the clinical treatment response to modern therapeutic methods including dupilumab, JAK inhibitors and topical corticosteroids. Our work provides an initial framework for understanding pharmacogenetic determinants of treatment response in AD, informing future personalized approaches for disease management.

## 2. Materials and Methods

### 2.1. Patient Recruitment

We conducted a multicentric pharmacogenetic study in three major dermatology outpatient clinics in Greece, including the Dermatology Clinics of the “Andreas-Syggros” Hospital of Athens, the University General Hospital of Larissa and the Dermatology Clinic of the Medical School of the University of Patras. Patients of Greek origin with clinically diagnosed AD receiving targeted therapy were prospectively recruited during clinical routine care from inception to September 2023. Therapeutic regimens included the monoclonal antibody dupilumab, topical corticosteroids (TCs), and JAK inhibitors such as abrocitinib, baricitinib, and upadacitinib. Baseline demographic data including age, sex, weight, height, smoking status and disease-relevant parameters such as disease onset and disease duration were recorded at first visit. Disease severity was assessed using the Eczema Area and Severity Index (EASI), ranging from 0 to 72, with higher scores indicating greater disease severity. Treatment response was assessed through the change in the EASI score during a follow-up visit 4 months ±2 weeks after treatment initiation.

All participants provided written informed consent prior to enrollment. The study was conducted in accordance with the Declaration of Helsinki and approved by the relevant institutional ethics committees.

### 2.2. Genomic DNA Isolation and Genotyping

Peripheral blood samples were collected from a total of 43 Greek patients with AD in K3-EDTA tubes stored at −20 °C at the first visit. Genomic DNA (gDNA) was isolated with conventional methods using 500 µL of whole blood and the PureLink™ Genomic DNA Mini Kit (Thermo Fisher Scientific, Waltham, MA, USA) using 200 µL of whole blood following the manufacturer’s protocol. In both cases, quantification and integrity of the isolated gDNA were assessed through spectrophotometry and agarose gel electrophoresis. Genotyping of the polymorphisms under study, *IL6R* rs2228145 A>C, *TRAF3* rs12147254 G>A and *JAK1* rs2780815 T>G were performed through the polymerase chain reaction-restriction fragment length polymorphism (PCR-RFLP) method using allele-specific restriction enzymes.

Target regions encompassing each polymorphism were amplified using PCR with 50 ng of genomic DNA per reaction. Primers were designed using the Primer3 algorithm [25]. Primer sequences, melting temperatures (Tm), and amplicon sizes are provided in Table 1. Each PCR reaction consisted of 1U Taq polymerase, 1× buffer solution with 1.5 mM MgCl_2_, 0.1 mM dNTP mix and 0.1 μM of each primer at a final reaction volume of 20 µL. The PCR protocol for the *IL6R* rs2228145 A>C polymorphism included pre-incubation at 95 °C for 5 min, followed by 32 cycles of denaturation at 95 °C for 30 s, annealing at 63 °C for 30 s and extension at 72 °C for 45 s. For *TRAF3* rs12147254, the protocol included pre-incubation at 95 °C for 5 min, followed by 35 cycles of denaturation at 95 °C for 45 s, annealing at 59 °C for 45 s and extension at 72 °C for 45 s. For the *JAK1* rs2780815 T>G variant, pre-incubation was performed at 95 °C for 5 min, followed by 35 cycles of denaturation at 95 °C for 30 s, annealing at 60 °C for 30 s and extension at 72 °C for 30 s. All PCR reactions were concluded by a final extension at 72 °C for 5 min. Amplification programs were conducted using the C1000 Touch Thermal Cycler (BIO-RAD, Hercules, CA, USA). Qualitative analysis of the PCR products was performed on a 1.5% *w*/*v* agarose gel through electrophoresis.

The amplified PCR products were then subjected to PCR-RFLP analysis. The *IL6R* rs2228145 A>C polymorphism was genotyped using two units of the *HindIII* restriction enzyme at a final reaction volume of 20 µL. Similarly, the *TRAF3* rs12147254 G>A variant was genotyped with two units of the *BssSI* enzyme per 20 µL reaction, while two units of the *Sm1I* restriction enzyme per 20 µL reaction were employed for the *JAK1* rs2780815 T>G SNP. All reactions were incubated overnight at a heating block using 37 °C for *HindIII* and *BssSI* and 55 °C for *Sm1I*. Fragment separation of the digested PCR products was performed using electrophoretic analysis in a 3% *w*/*v* agarose gel. At the *IL6R* region, the presence of the common A allele presents a recognition site, leading to the production of two DNA fragments of size 447 and 99 bp, while in the presence of the rare C allele it creates a second recognition site, resulting in the production of three DNA fragments of length 265, 182 and 99 bp. For *TRAF3*, the presence of the common G allele presents a single recognition site, leading to the production of two DNA fragments of size 173 and 100 bp. For the *JAK1* variant, the presence of the common T allele presents one recognition site, leading to the production of two DNA fragments of size 302 and 73 bp. On the other hand, the presence of the rare G allele creates a second recognition site, resulting in the production of three DNA fragments of length 250, 73 and 52 bp. A representative image of expected fragments is presented at Figure S1.

### 2.3. Statistical Analysis

Continuous variables are presented as median and interquantile ranges (IQR), while categorical variables are summarized as counts and percentages. The primary outcomes were absolute change in Eczema Area and Severity Index (ΔEASI), quantified as ${EASI}_{final}-{\Delta EASI}_{baseline}$, and percentage change (ΔEASI%), calculated as $\;\Delta EASI\%=100\times \frac{{EASI}_{baseline}-{EASI}_{final}}{{EASI}_{baseline}}$. The chi-square test was used to test for Hardy–Weinberg equilibrium. Comparisons across treatment groups were conducted with the Kruskal–Wallis test for continuous variables and Fisher’s exact test for categorical variables.

To evaluate the extent of the effect of each genetic variant to the absolute (ΔEASI) and relative (ΔEASI%) treatment response scores, multivariable linear regression models were used. Variant allele information was encoded under an additive genetic model based on the number of minor alleles. Analyses were initially performed in the full cohort, followed by stratified analyses within each treatment group. Statistical models using all drugs were adjusted for age, sex, body mass index (BMI), smoking status, baseline EASI, disease onset and the drug administered. For stratified analyses, the drug term was excluded, and analyses were restricted to drugs administered to ≥5 patients. Regression coefficients, standard errors and *p*-values are reported for the genetic term.

As a secondary outcome, we assessed the association between response to treatment represented as a binary outcome and allele frequencies of the genotyped SNPs through non-parametric tests. Patients were defined as responders when achieving an at least 75% improvement in EASI score (ΔEASI ≥ 75%) after 4 months (±2 weeks) of therapy. The 2 × 2 contingency tables were used to examine potential associations. Cochran–Armitage trend test, allelic and genotypic tests, and tests of dominant or recessive SNP inheritance were utilized to determine genotype associations with therapeutic response. The threshold for all statistical tests was set at *p*-value ≤ 0.05. All statistical analyses were performed in R v4.3.3 (R Foundation for Statistical Computing, Vienna, Austria).

## 3. Results

In total, 43 Greek patients suffering from AD with complete clinical and genotypic data receiving treatment were included in our study. Table 2 summarizes the demographic and clinical characteristics of the study population. The median age of patients was 35 (28–54) years, where 21 (47.7%) were male. The median body mass index was 25.35 (23.08–28.29) kg/m^2^, while 17 (38.6%) were self-reported as smokers. The median disease duration was estimated at 15 (7–25) years, with a baseline EASI at 15 (7.4–22.5). This was decreased to −9 (−17 to −3) following 4 months of therapy, nevertheless displaying variability in treatment response across individuals and treatments groups as observed elsewhere [15].

Among these, 19 patients received treatment with dupilumab, while 14 patients received treatment with small molecules targeting the JAK/STAT pathway, specifically abrocitinib (n = 3), upadacitinib (n = 10) and baricitinib (n = 1), while 10 patients received TCs. In subgroup analyses, clinical characteristics were largely comparable across treatment groups, although variability in sample size was observed. The only exception was observed in age, where patients with dupilumab were older compared with those treated with JAK inhibitors or TCs (*p*-value = 0.047; Table 2). Patients treated with dupilumab had a median age of 52 (36.5–56.5) years (10 males, 52.63%) and a median baseline EASI of 16 (6.4–24.5), while those receiving JAK inhibitors (n = 14; median age: 30.5 (24.5–48.25) years) showed a median baseline EASI of 8.6 (4–19.5). Patients treated with TCs demonstrated a median baseline EASI of 17 (10.5–21.25). Trajectories of the EASI score 4 months ± 2 weeks after treatment administration stratified by drug are shown at Figure S2.

### 3.1. Association of JAK/STAT Variants and Treatment Efficacy

All of the analyzed variants were consistent with the Hardy–Weinberg equilibrium (*p*-value*_TRAF3_* = 0.35, *p*-value*_JAK1_* = 0.979, *p*-value*_IL6R_* = 0.589). Multivariate linear regression analyses were performed to evaluate the association between our selected JAK/STAT-related variants and treatment response, as measured by absolute (ΔEASI) and relative (ΔEASI%) change in the baseline EASI score. Models incorporating all treatment regimens were adjusted for age, sex, body mass index, smoking status, baseline EASI, disease duration and administered drug under an additive genetic model.

**Figure 1:**
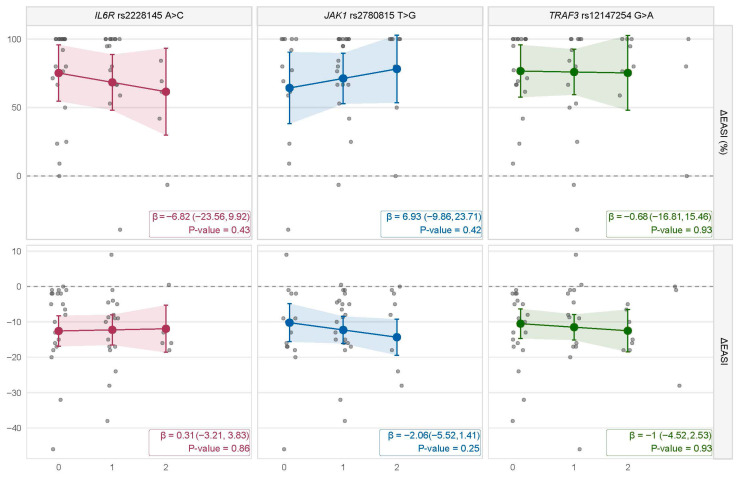
Pharmacogenetic associations of *IL6R*, *JAK1* and *TRAF3* genotypes with ΔEASI and ΔEASI% change in atopic dermatitis. The panels display adjusted changes in Eczema Area and Severity Index (ΔEASI, absolute and percentage) according to minor allele count for each gene. Colored lines represent predicted ΔEASI values from linear regression models adjusted for age, sex, BMI, smoking status, baseline EASI, and disease onset. Shaded ribbons report 95% confidence intervals. Individual patient ΔEASI values are shown as gray dots (jittered horizontally for clarity). Numerical annotations indicate the regression coefficient (β), 95% confidence interval, and *p*-value.

When evaluating the absolute ΔEASI change, baseline EASI was consistently the strongest predictor of treatment response (beta ~ −0.65 to −0.69, *p*-value < 10 × 10^−7^), suggesting that patients with higher baseline disease activity experienced greater absolute reductions in EASI (Table S1). This is somewhat expected since the absolute change is inherently dependent on baseline values, reflecting greater potential for improvement among patients with more severe AD. None of the other covariates were significantly associated with ΔEASI in the overall cohort (Table S1). Similarly, allelic effects of *TRAF3* rs12147254 G>A, *JAK1* rs2780815 T>G and *IL6R* rs2228145 A>C variants overlapped the null (Figure 1). Similarly, null effects were retrieved when assessing the relative ΔEASI% change, where no significant associations were retrieved for the overall cohort (Figure 1; Table S1).

When conditioning on each specific treatment, no significant pharmacogenetic association was observed among patients receiving dupilumab (n = 19; Table S2), with all tested variants demonstrating null effects on both absolute and relative ΔEASI outcomes. In contrast, within the upadacitinib-treated subgroup (n = 10), the minor allele of the *IL6R* rs2228145 variant exhibited a negative association with percentage change in EASI (β = −30.7 ± 5, *p*-value = 0.026), suggesting that each additional copy of the minor allele was associated with a reduced relative treatment response (Table S2). However, given the small sample size of subgroup analyses (n ranging from 10 to 19), these findings should be interpreted as exploratory.

### 3.2. Association of JAK/STAT Variants and Response Status

We further evaluated the association between genotyped variants and binary response status, determined through at least 75% improvement in EASI score after 4 months ±2 weeks of therapy. None of the examined SNPs reported significant associations across the cohort (n = 43). However, the *IL6R* rs2228145 A>C variant showed a nominal odds ratio of 8.66 (95% confidence intervals: 0.87–86.6; *p*-value = 0.065) (Table 3). Similar results were identified when stratifying by specific pharmacotherapies, where results overlapped the null (Table S3).

## 4. Discussion

Here, we present the first pharmacogenetic investigation of treatment response in AD, focusing on genetic variants within the IL6/JAK/STAT signaling axis. Despite the established role of this pathway in AD pathophysiology serving as a direct pharmacological pathway in modern therapeutic approaches, our results indicate an overall lack of robust pharmacogenetic effects across the examined variants in the full cohort. Nevertheless, we identify suggestive, upadacitinib-specific signals that warrant further investigation.

The absence of significant associations in the overall cohort suggests that common variants within *IL6R*, *JAK1*, and *TRAF3* do not exert a major, treatment-agnostic effect on clinical response, at least within the limits of this sample size. AD represents a highly heterogeneous disease, both at the biological and clinical level, with multiple partially overlapping inflammatory endotypes. While the JAK/STAT pathway is central to cytokine signaling, its contribution is context-dependent and interacts with parallel axes, including IL-4/IL-13-driven type 2 inflammation, barrier dysfunction, and environmental triggers. As such, single-variant effects are likely attenuated when evaluated across heterogeneous therapeutic modalities and patient subgroups. This is further supported by the consistent observation that baseline EASI score was the strongest predictor of treatment response in the absolute ΔEASI models, reflecting the greater room for absolute improvement of clinical scores in patients with higher baseline disease activity exhibiting greater absolute improvement.

Our stratified analyses further provide a more refined view of treatment-specific pharmacogenetic effects. No significant associations were derived for topical corticosteroids nor the monoclonal antibody dupilumab (Table S2), where the latter is consistent with findings from genome-wide approaches reporting limited genetic background in treatment response with biological approaches [26]. However, a significant association between the *IL6R* rs2228145 A>C variant and reduced percentage improvement in ΔEASI% was observed in the upadacitinib subgroup. Specifically, the minor allele was associated with attenuated treatment response (Table S2). While clearly exploratory due to the small sample size, this finding is biologically plausible and aligns with the functional role of *IL6R* in modulating downstream JAK/STAT signaling. Specifically, the rs2228145 variant is an exonic, non-synonymous common SNP (Asp358Ala) that occurs within the region encoding the proteolytic cleavage site affecting shedding [27]. This leads to differences in IL6R concentrations between carriers of different alleles, with reduced concentrations of membrane-bound IL6R and increased concentrations of soluble IL-6R in carriers of the minor C allele [28]. This allele has been further associated with a variety of common diseases across different clinical spectra, such as asthma [29], amyotrophic lateral sclerosis progression [30] and coronary artery disease [31].

Analysis of treatment response as a binary outcome yielded largely consistent results, with no significant associations across variants. Integrating both approaches enabled us to (i) minimize loss of statistical power when dichotomizing continuous variables [32], that is, treatment response quantified through clinical scores, and (ii) evaluate both the effect of genetic variation in treatment efficacy as well as treatment response. However, we observed a nominally significant role for the *IL6R* rs2228145 A>C variant and treatment response in our full cohort. Specifically, the presence of the *IL6R* rs2228145 A allele demonstrated a notable effect size showcasing eight-fold increased odds of response, albeit with wide confidence intervals and nominal statistical significance (*p*-value = 0.065; Table 3). Despite being non-significant, this finding strengthens the role of *IL6R* genetic variation in treatment response, implying the need for validation in larger cohorts, a goal we are poised to pursue.

Several limitations must be considered when interpreting these findings. To begin with, the limited sample size incorporated in our study reduces statistical power and increases the likelihood of both type I and type II errors, particularly in treatment-stratified analyses. This is especially relevant given the modest effect sizes expected for common variants in complex traits, as is treatment response. In addition, despite the robust usage of PCR-RFLP genotyping, this methodology restricts our investigation to a small number of candidate variants. To mitigate this, we selected variants with relatively high minor allele frequency (MAF) in our candidate gene approach as estimated through the 1000 Genomes Project, European reference panel (*IL6R* rs8192284, MAF = 0.39; *TRAF3* rs12147254, MAF = 0.3; *JAK1* rs2780815, MAF = 0.46) [33]. Hence, we increased the likelihood of detecting at least a single copy of a minor allele in our under-study cohort. In addition, we note that certain potentially relevant clinical variables for treatment response, such as alcohol consumption and vitamin D levels, were not consistently available at the individual level and were therefore not included in the analysis. However, our pharmacogenetic analyses were adjusted for age, sex and relevant clinical covariates including disease activity and BMI, a known risk factor for AD [34] and other skin diseases [35], thereby partially reducing the potential confounding arising from baseline clinical heterogeneity. Beyond these methodological caveats, a broader conceptual limitation lies in the candidate gene approach itself. While biologically informed and useful in eczema research [36], this strategy assumes prior knowledge of relevant pathways and may overlook important genetic determinants outside the selected axis [37]. Emerging evidence from other inflammatory diseases suggests that pharmacogenetic effects are often polygenic and distributed across multiple pathways, including immune regulation, drug metabolism, and tissue-specific factors [38]. As such, genome-wide approaches or integrative multi-omic strategies may be required to fully capture the complexity of treatment response in AD.

Despite these limitations, our study provides a proof-of-concept for pharmacogenetic investigations in AD and highlights several important considerations for future research. First, we provide evidence for the employment of pathway-specific genetic effects in selected treatment modalities, particularly when evaluating targeted therapies with distinct mechanisms of action. Second, the observed signal at *IL6R* likely suggests upstream cytokine signaling variation influencing JAK inhibition as a therapeutic approach, a hypothesis that can be directly tested in larger, independent cohorts. Third, the integration of genetic data with clinical and molecular phenotyping may enhance the identification of biologically meaningful subgroups, moving toward a more precise stratification of patients.

## 5. Conclusions

To our knowledge, we present the first pharmacogenetic study in AD in a cohort of 43 Greek patients. Although no robust pharmacogenetic associations were identified at the cohort level, our findings suggest that genetic variation within the IL6 signaling axis may modulate response to JAK inhibition in a treatment-specific manner. These results should be considered exploratory but also provide a biologically grounded framework for future studies aiming to investigate the genetic variation in therapeutic response in AD.

## Figures and Tables

**Table 1:** Primer sequences, PCR product size and Tmelting temperature (Tm).

	Primer Sequence	Tm	PCR Product
*IL6R* rs2228145			
Forward primer	5′-CCCTGAGCTTGAGGTGTCTC-3′	61.4 °C	546 bp
Reverse primer	5′-CACCTAAAACACGGCTTGGC-3′	59.4 °C	
*TRAF3* rs12147254			
Forward primer	5′-AAGAGTCTGGTGGCATTGGG-3′	59.9 °C	273 bp
Reverse primer	5′-GCTAACACTTGGGCTAGGCT-3′	59.7 °C	
*JAK1* rs278081			
Forward primer	5′-TACCCAGTGCTACCCCACTT-3′	59.4 °C	375 bp
Reverse primer	5′-GCCTCTGACGTCTGGTCTTT-3′	59.4 °C	

**Table 2:** Clinical and demographic characteristics of 43 patients with atopic dermatitis stratified by treatment group. Continuous variables are presented as median (interquartile range, IQR), and categorical variables as counts (percentages). Comparisons between groups were performed using the Kruskal–Wallis test for continuous variables and Fisher’s exact test for categorical variables. Significant differences are denoted with bold font.

Variable	Total (n = 43)	Dupilumab (n = 19)	JAK Inhibitors (n = 14)	Topical Corticosteroids (n = 10)	*p*-Value
Age, years	35 (28–54)	52 (36.5–56.5)	30.5 (24.5–48.25)	33.5 (23.25–41)	**0.047**
Sex (male) (%)	21 (47.7%)	10 (52.63%)	6 (42.85%)	5 (50%)	0.924
Body mass index, kg/m^2^	25.35 (23.08–28.29)	25.35 (23.53–29.34)	25.64 (22.33–28.62)	25.36 (22.38–27.09)	0.788
Smoking (yes/no) (%)	17 (38.6%)	10 (52.63%)	3 (21.42%)	4 (40%)	0.178
Disease duration, years	15 (7–25)	8.00 (4–15.5)	21.5 (13.5–27.5)	15.5 (11.5–25)	0.073
Baseline EASI	15 (7.4–22.5)	16 (6.4–24.5)	8.6 (4–19.5)	17 (10.5–21.25)	0.436
Follow-up EASI	2 (0–6)	3 (0–8.15)	0.5 (0–3.5)	2 (0.25–5)	0.458
ΔEASI (absolute)	−9 (−17 to −3)	−8 (−16 to −1.5)	−6.85 (−15.25 to −2)	−16 (−17.75 to −9.25)	0.208
ΔEASI (percentage)	84.21 (64.1–100)	84.21 (37.5–100)	97.5 (67.86–100)	80 (76.67–98.68)	0.693
Reponders (%)	17 (38.36%)	10 (52.63%)	9 (64.28%)	8 (80%)	0.373

**Table 3:** Pharmacogenetic association analysis between treatment response and the under-study genetic variants in atopic dermatitis.

Model	Responders	Non-Responders	Odds Ratio	95% CI	*p*-Value
*TRAF3* rs12147254 G>C					
Genotypic (GG/GA/AA)	11/10/6	8/7/1	N.A.	N.A.	0.389
Cochran–Armitage (GA)	32/22	23/9	0.5691	0.22–1.46	0.241
Dominant (GG+GA/AA)	21/6	15/1	0.233	0.025–2.144	0.198
Recessive (GG/GA+AA)	11/16	8/8	0.687	0.197–2.387	0.555
*JAK1* rs2780815 T>G					
Genotypic (TT/TG/GG)	8/13/6	6/8/2	N.A.	N.A.	0.701
Cochran–Armitage (T/G)	29/25	20/12	0.696	0.28–1.7	0.4266
Dominant (TT+TG/GG)	21/6	14/2	0.5	0.087–2.841	0.434
Recessive (TT/TG+GG)	8/19	6/10	0.701	0.19–2.591	0.595
*IL6R* rs2228145 A>C					
Genotypic (AA/CA/CC)	14/12/1	7/5/4	N.A.	N.A.	0.701
Cochran–Armitage (A/C)	40/14	19/13	1.954	0.769–4.963	0.158
Dominant (CC+CA/AA)	26/1	12/4	8.66	0.872–86.06	0.065
Recessive (CC/CA+AA)	14/13	7/9	1.384	0.399–4.8	0.607

Abbreviations: CI, confidence intervals; N.A., not applicable.

## Data Availability

All data are available from the corresponding author upon reasonable request.

## Supplementary Materials

The following supporting information can be downloaded at: https://www.mdpi.com/article/10.3390/genes17050575/s1, Figure S1. Representative image of PCR-RFLP electrophoretic analysis results. Figure S2. Trajectories of the Eczema Area and Severity Index (EASI) score after 4 months ± 2 weeks after therapy, stratified by administered drug. Table S1. Covariate association statistics. Table S2. Drug-subgroup pharmacogenetic analysis results under a linear regression model. Table S3. Drug-subgroup pharmacogenetic analysis results under 2 × 2 contingency tables.
