# Supplementary material for: Pharmacogenetic Analysis of Variants in IL-6 Signaling and Response to Modern Therapeutic Approaches in Greek Patients with Atopic Dermatitis
Source: Genes (Basel). 2026 May 18;17(5):575. doi: 10.3390/genes17050575 (PMC13205716; doi:10.3390/genes17050575)

Figure S1. Representative image of PCR-RFLP electrophoretic analysis results.

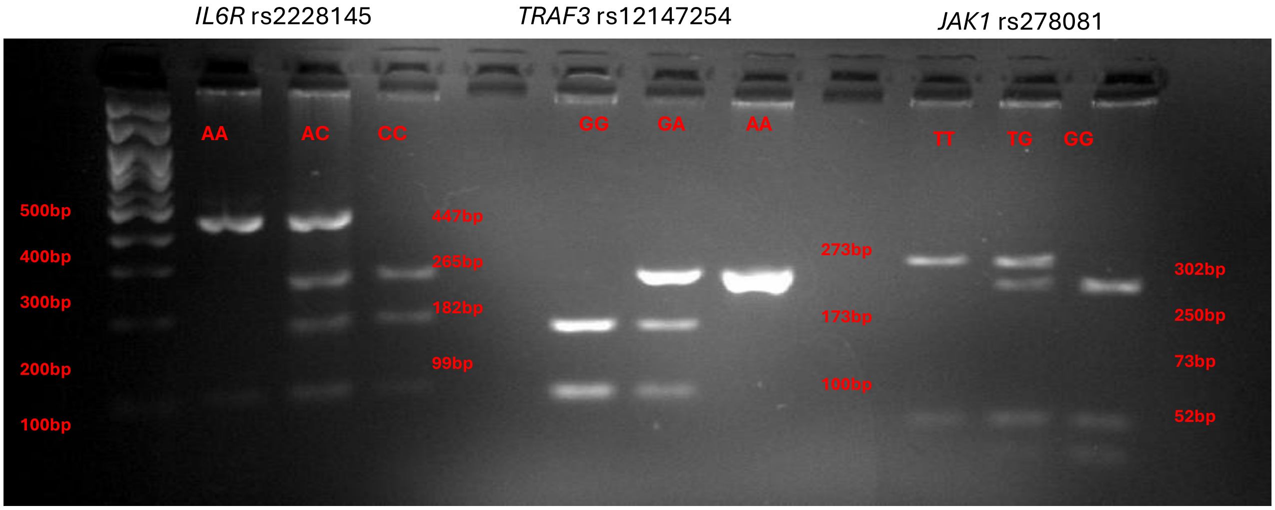

Figure S2. Trajectories of the eczema area and severity index (EASI) score after 4 months ± 2 weeks after therapy, stratified by administered drug.

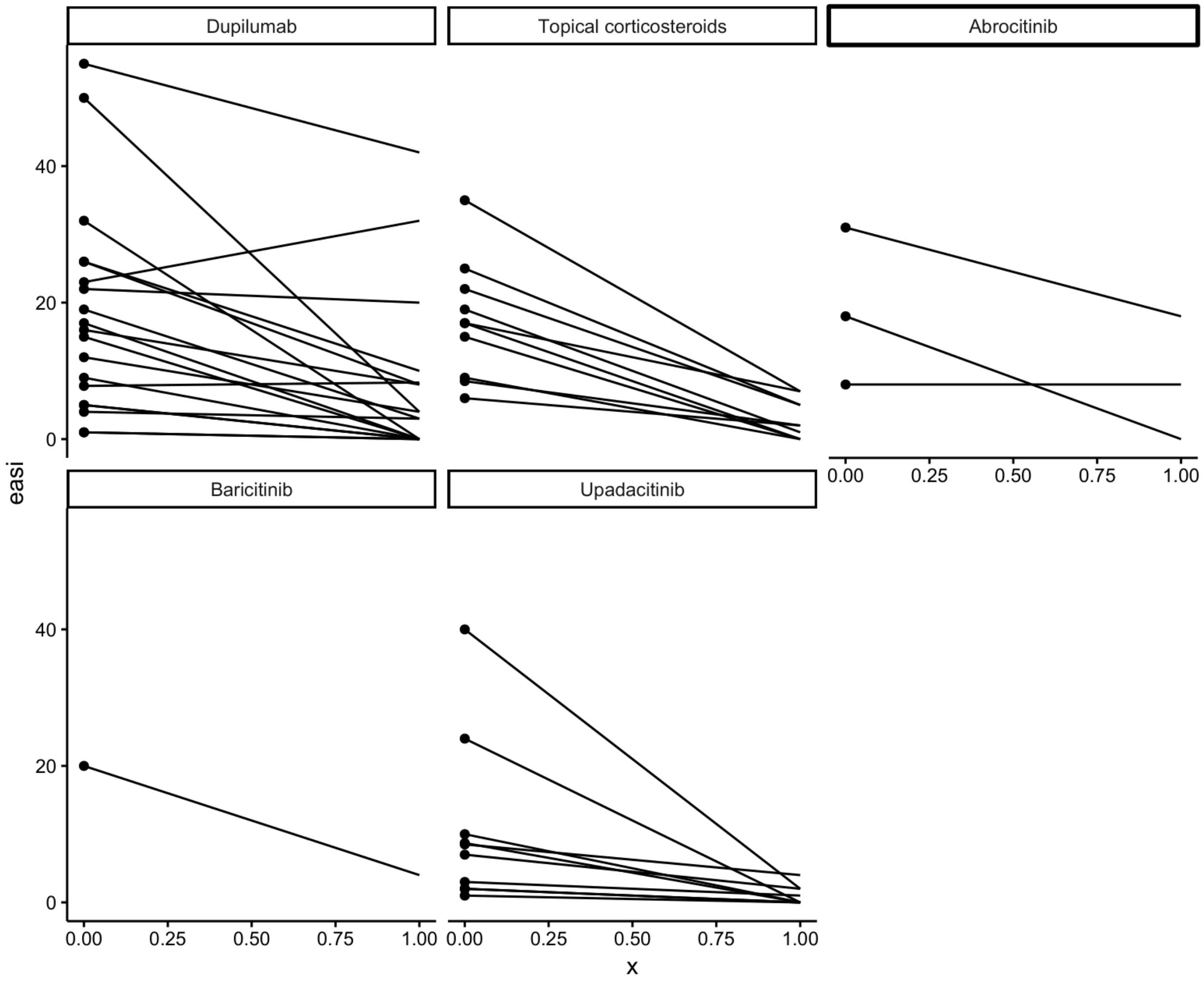

Supplement: Supplementary file 1 [file genes-17-00575-s001.zip › Supplementary_figures.pdf]
